# Safeguarding palliative and serious illness care in America

**DOI:** 10.1016/j.lana.2026.101584

**Published:** 2026-07-31

**Authors:** William E. Rosa, Gina Piscitello, Diane E. Meier, Arif H. Kamal, Jean S. Kutner, Abby R. Rosenberg, Allison Silvers, Stacie Sinclair, Robert M. Arnold

**Affiliations:** a Department of Psychiatry and Behavioral Sciences, Memorial Sloan Kettering Cancer Center, New York, NY, United States of America; b Section of Palliative Care and Medical Ethics, University of Pittsburgh, Pittsburgh, PA, United States of America; c Department of Geriatrics and Palliative Medicine, Icahn School of Medicine at Mount Sinai, New York, NY, United States of America; d Center to Advance Palliative Care, Icahn School of Medicine at Mount Sinai, New York, NY, United States of America; e American Cancer Society, Kennesaw, GA, United States of America; f Department of Medicine, Duke University School of Medicine, Durham, NC, United States of America; g American Academy of Hospice and Palliative Medicine, Schaumburg, IL, United States of America; h Department of Medicine, University of Colorado Anschutz School of Medicine, Aurora, CO, United States of America; i Department of Supportive Oncology, Dana-Farber Cancer Institute, Boston, MA, United States of America; j Department of Pediatrics, Boston Children's Hospital, Boston, MA, United States of America; k Department of Pediatrics, Harvard Medical School, Boston, MA, United States of America

**Keywords:** Palliative care, Hospice, Serious illness, End of life, Pain management, Privatization, Research and development, Health policy

If a society's greatness is measured by how it treats its most vulnerable, we live in discouraging times for the more than 13 million adults and 700,000 children with serious illnesses in the United States of America (U.S.).^1^^,^^2^ While progress had been made to improve serious illness care over the past few decades, emerging policies and practices threaten the important role that palliative care plays in the improvement of quality patient care and outcomes throughout the disease continuum and especially at the end of life. In this personal view, we describe three threats to specialty palliative care in the U.S., namely healthcare financing changes, increased privatization of services, and low prioritization of palliative care research in myriad contexts, including pharmaceutical and clinical trial research and development. We subsequently provide guidance for multi-sector actors to address these threats and mitigate harms while optimizing palliative care for U.S. populations.

Although seminal international reports suggest that high-income nations, such as the U.S., lead the way in palliative care availability and access, diverse and complex challenges persist.^3^, ^4^, ^5^, ^6^ The Institute of Medicine's (IOM's) 2014 *Dying in America* report as well as subsequent research has found that people living with serious illness are experiencing potentially avoidable suffering and receiving treatments that may lack benefit or are misaligned with their goals.^7^ For example, symptoms are commonly under-treated; despite encouraging trends of decreased pain prevalence and severity, more than 30% of patients with metastatic cancer still report moderate to severe levels of pain.^8^ Shared decision-making and conversations on treatment preferences happen less than half the time^9^; although most U.S. adults prefer to die at home, one-third of deaths still occur in U.S. hospitals^10^^,^^11^ and the place of death for children with serious illness follows similar trends.^12^ And high caregiver burden is associated with both increased caregiver mortality and the placement of patients with serious illness in long-term care facilities.^13^^,^^14^

While an extensive literature supports specialty palliative care integration for several serious illnesses, including cancer, pulmonary, renal, and liver diseases, heart failure, and in neurology^15^, ^16^, ^17^, ^18^, ^19^, ^20^, ^21^; suboptimal outcomes are concentrated among the most vulnerable Americans across diseases. A cohort study of 8976 nationally representative decedents ≥65 years of age who died during 2000–2021 demonstrated that lower wealth was associated with a significantly higher symptom burden, partially mediated by higher multimorbidity, functional impairment, and dementia rates.^22^ Moreover, multiple studies have identified Black and Hispanic adult patients are less likely to receive medically indicated opioid pain medications at the end-of-life when compared to White patients.^23^, ^24^, ^25^ Children are also at high risk for poor serious illness outcomes. Studies have reported worse end-of-life pain-control outcomes among Hispanic children^12^ and a review of children's hospital association benchmark reports found that 55% report not having access to hospice care, with 79% reporting no access to respite care for caregivers.^26^

## Strengths and shortfalls of palliative care in the U.S.

**Fig. 1:**
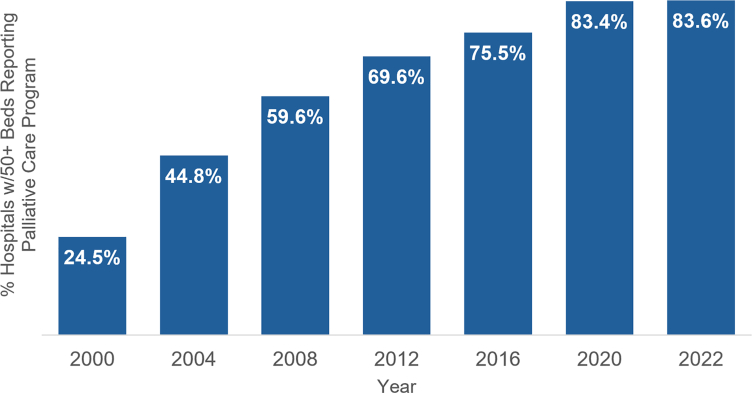
**Palliative care programs in U.S. Hospitals with 50 or more beds, 2000–2022**.

Source: Center to Advance Palliative Care. Used with permission.

The growth of specialty palliative care across the U.S. over several decades improved the overall quality of care of patients with serious illness, access to critical symptom management interventions and services, and cost-savings.^27^ Specialty palliative care teams–often consisting of interdisciplinary professionals that include physicians, advanced practice providers, nurses, social workers, chaplains, and sometimes pharmacists and recreational therapists—aim to provide holistic, patient- and family-centered care in hospital and outpatient settings.^28^ These teams are reported at most U.S. adult hospitals (>84% of hospitals with 50 or more beds, >96% of hospitals with greater than 300 beds), up from only 50% of hospitals in 2014.^29^ Fig. 1 shows the proportion of U.S. hospitals with 50 or more beds with palliative care programs from 2000 to 2024. While only 35% of rural hospital report a palliative care program, the rise in telehealth provides new opportunities to provide palliative care in these settings.^30^ In addition, most children's hospitals have specialty palliative care teams, especially at freestanding hospitals that provide high acuity pediatric care.^26^

Self-identified home-based palliative care services are now available in about 80% of U.S. counties, and an increasing number of Medicare Advantage plans offer home-based palliative care as supplemental benefits (2% in 2020, 4% in 2024).^31^^,^^32^ Policy support, new reimbursement opportunities for primary and specialty palliative services, and federally-funded research strengthen the ability for palliative care to be provided to patients with serious illness. For example, national professional organizations, including the American Society of Clinical Oncology (ASCO), the Oncology Nursing Society, the American Academy of Pediatrics, the American College of Cardiology, and the Renal Physicians Association recommend specialty palliative care for people of any age with advanced illness.^15^^,^^33^, ^34^, ^35^, ^36^, ^37^ In addition, the National Institutes of Health is increasingly supportive of palliative care research.^38^^,^^39^ In 2025, the Advancing the Science of Palliative Care Research Across the Lifespan (ASCENT) Consortium was awarded a 5-year $64 million grant, supported by six National Institute of Health (NIH) institutes, to support research focused on improving the quality of life for people living with serious illness across diverse diagnoses and ages.

Despite these improvements, the U.S. health system is still not optimized to care for people with serious illness and those who care for them. A recent retrospective cohort study of 33,744 fee-for-service Medicare decedents ≥66 years of age with advanced cancer found persistent patterns of people receiving care that is expensive and unlikely to improve quality of life or survival.^40^ There are still high rates of emergency department visits and unplanned hospitalization near death, low uptake of palliative care early in patients’ disease course, and late hospice enrollment, with a median hospice length of stay of just 18 days despite hospice being a healthcare benefit for patients with a prognosis of six months or less.^41^ While most inpatients have access to specialty palliative care, this is not the case for outpatients. For example, only 22% of patients with metastatic breast cancer were seen in ambulatory palliative clinic at one comprehensive cancer center, and fewer than the 37% were seen by inpatient palliative care.^42^ Access is also not equitable across the U.S.—the available of specialty trained palliative care professionals per 100,000 population varies from 10.3 in the top U.S. state with capacity to deliver high-quality care to patients with serious illness to only 2.4 in the bottom state.^31^ Differential access to adequate pain management also continues to be a persistent problem. For example, data suggests the 2016 Centers for Disease Control and Prevention guideline for prescribing opioids may have had the unintended effect of decreasing opioid prescriptions for patients with serious illness and increasing health care resource use.^43^^,^^44^

## Threats to palliative care in the U.S.

Ongoing changes in health care financing threaten the advances of both specialty palliative care and hospice care. Specialty palliative care provided by physicians and advanced practice providers is supported by most U.S. insurance plans for patients with serious illness receiving care in settings such as hospitals, clinics and home. The financial incentive for health systems to incorporate specialty palliative care is often cost-savings, because low reimbursement from insurance payers limits revenue generation from the provision of these services.^45^ Hospice care in the U.S. is a Medicare insurance benefit that, unlike specialty palliative care, requires a prognosis of six months or less. While Medicare Part A may cover 100% of hospice costs including medical care and equipment, hospice patients with private insurance may have additional costs.

The first threat is a sustained escalation in profit-driven healthcare. Over the past 40 years, many healthcare organizations including hospitals, nursing facilities, and hospice agencies have been purchased by publicly-traded companies and private equity investors. Thirty-six percent of all U.S. hospitals are for-profit, up from only 13% in 2000, while 43% of U.S. nursing homes are owned by for-profit chains, with another 15% owned by private equity and real estate investment trusts.^46^^,^^47^ While providing capital for potential improvement, data suggest these changes lead to a decline in various hospital measures of quality and safety, increased nursing home mortality, poorer quality of care near the end of life, and decreased patient satisfaction.^48^ For-profit hospices have grown to over 70% of the market despite data showing lower caregivers’ ratings, lower number of staff per patient, and higher rates of patient disenrollment than not-for-profit hospices.^48^^,^^49^ There are stark differences between for-profit and not-for-profit health care organizations in terms of access to specialty palliative care. Only 49% of for-profit hospitals in the U.S. report providing specialty palliative care and there is some indication that for-profit hospices are less likely to support non-hospice palliative care programming (e.g. home visits for symptom management for patients with greater than six months prognosis).^31^

Second, greater privatization may irreparably disrupt the growth of palliative care as investors focus primarily on drug development and treatment-focused innovation, potentially resulting in less quality care for people with serious illness and at the end-of-life.^49^^,^^50^ As health services are privatized, access to holistic care for people living with complex symptom burdens may erode in a financialized setting that must produce strong quarterly earnings. Thus, continued financial and policy investment in palliative care is essential for innovation. For example, ASCO recommends specialty palliative care referral for patients on early phase clinical trials because it helps them maintain quality of life and functional independence while potentially bolstering participant engagement, retention, and treatment adherence.^15^ Although serious illness care and research–comprised largely of pain, symptom, and psychosocial management and interventions–cannot be configured into a pill, it is essential to improve care quality and must be emphasized across care delivery settings, including clinical trials.

Third, palliative care research in areas including drug and device development and health services research is not currently prioritized by organizations including pharmaceutical companies, nor private foundations, despite the pressing need to advance the delivery of quality care to patients with serious illness and those who care for them. For example, from 2018 to 2022, only 0.2% of NIH research awards were related to palliative medicine.^38^ Widespread commitment to palliative care research beyond the previously mentioned ASCENT Consortium through the NIH mentioned earlier will be required for future scaling and sustainability of efforts.

Research has shown the impact of palliative care interventions in several practice settings and contexts, yet funding to support 1) dissemination and implementation research and 2) policy and payment model changes that facilitate implementation of care delivery models demonstrating effectiveness are both required to implement and scale evidence-based interventions to ensure scientific advances reach patients.^51^^,^^52^ Yet the lack of federal funding to implement new palliative care innovations limits their access. The 2025 U.S. federal government shutdown abruptly ended telehealth benefits for patients receiving Medicare limiting a programmatic change that had increased access to specialty palliative care (although many telehealth flexibilities have now been temporarily extended through 2027).^53^^,^^54^ Interventions like telehealth in the palliative care context demonstrate effectiveness but still require study and refinement, which cannot occur if federal policy does not support its use and privatization does not incentivize implementation and healthcare delivery science in serious illness care.^55^

These issues will worsen palliative care access and quality for our sickest patients and their families. The tenuous financial states of hospitals put specialty palliative care programs at risk, given their focus on value optimization and patient experience, not revenue generation. In turn, cuts to inpatient palliative care programs may increase length-of-stay and limit safe discharge options, while exacerbating non-beneficial and unnecessary overtreatment amid a specialty palliative care workforce shortage.^56^ More than one-third of U.S. home-based palliative care programs are supported by hospitals and face significant threat when resources tighten.^57^ The loss of specialty palliative care services across settings, which are associated with improved care quality and cost-savings, will result in diminished quality and costlier care for the sickest 5% of Americans who drive over 50% of all health care spending.^58^

## Multi-sector recommendations to safeguard palliative care

Except for the U.S., nearly all high-income countries that see an uptrend of profit-driven practices and health privatization also have guaranteed universal coverage with substantial price and access regulation.^59^ Thus, threats to palliative care services in the face of profit-driven health care delivery may uniquely affect Americans, as the U.S. spends more on health care than other high-income countries and does not provide universal healthcare coverage.^60^ This may contribute to differences in palliative care development in private and public settings, as private settings may be more incentivized to pursue cost-saving care such as specialty palliative care than public settings. Yet, given the current U.S. political climate, including the strong lobbying influence of private equity firms and investors, prohibition of private equity ownership is an unlikely solution.^61^ Rather, U.S. health policy can be modified to encourage—or even require—specialty palliative care access. For example, several private health insurers have developed financial incentive programs for their network hospitals which encourage quality palliative care services to be available to their members^62^; and a similar financial incentive exists in the Medicare Hospital Inpatient Quality Reporting Program for certain geriatric skills and processes, known as the Age-Friendly Hospital Measure. A Medicare requirement for access to specialty palliative care services in large hospitals would not only benefit patients and families, but would also generate financial and patient experience benefits for the facility itself.

Targeted and specific payment models can also increase access to community-based palliative care services. In California, legislation requiring all Medicaid managed care organizations to cover palliative care services for specific beneficiaries resulted in substantial growth in palliative care capacity and patients served. In Hawai'i, the Medicaid state plan was amended to include community-based palliative care as a covered benefit, providing a standardized mechanism for access, at least for Medicaid beneficiaries. In lieu of sufficient federal support, we need other states to explore similar legislation or add specific palliative care benefits into their state Medicaid plans.^63^

In conjunction with pharmaceutical and clinical research and development, all drug development and clinical trials related to patients with serious illness must budget for specialty palliative care research and integration. Investigators should include palliative care experts in study designs for these trials, while research-granting foundations and industry can move to require palliative and supportive care considerations in study proposals. These adaptations will enhance the potential for successful trial outcomes while optimizing patient wellbeing. Additional recommendations for multi-sector actors are summarized in Table 1.

**Table 1:** Recommendations to improve palliative and serious illness care in America.

Congress	• Reintroduce and pass the Palliative Care and Hospice Education and Training Act (PCHETA). This will expand the pipeline of qualified professionals to care for the population with serious illness, raise awareness of palliative care, and improve funding for research specific to palliative care interventions. • Eliminate the geographic and originating site restrictions for Medicare reimbursement of telehealth visits. This will enable reliable revenue for efficient palliative care services delivery, particularly for patients who face hardships traveling to in-person visits.
Federal regulators	• Require patient reported symptom monitoring and other burden assessments in Medicare alternative payment models and Medicare shared savings programs. • Increase the value of the Medicare complexity add-on code in all settings, to ensure resources for multi-disciplinary care for complex patients. • Test a palliative care add-on code in Medicare.
State policy makers	• Pass legislation requiring Medicaid managed care organizations to cover palliative care services, or amend the Medicaid state plan to include community-based palliative care services. • Provide payment models to enable palliative care consultations to rural hospitals and federally qualified health centers. • Establish/expand loan forgiveness for palliative care professionals. • Support public awareness campaigns to educate the general public on palliative care and its benefits.
Private research foundations	• Encourage research trials that include people living with serious illness and address their needs. • Encourage research trials to measure quality of life and symptom burden in people living with serious illness, especially cancer, heart failure, advanced respiratory illnesses, advanced neurological illnesses, advanced kidney disease, and advanced liver disease. • Encourage research aimed at promoting patient quality of life and caregiver support.
Pharmaceutical companies	• Require companies engaged in clinical trial drug development for serious illnesses to offer accompanying specialty palliative care provision during trial delivery. • Expand research and development efforts to novel therapies for pain and other symptoms.
Hospital and health system leaders	• Ensure new, expanded, or continued availability of a multidisciplinary specialty palliative care team, sufficient in size to care for at least 5% of adult and pediatric inpatients. • Develop and implement processes to proactively identify patients in need of palliative care consultations, ideally in critical care, medical and surgical units, and in the emergency department. • Implement standardized processes to document goals of care discussions and advance directive documents in the electronic medical record. • Support interdisciplinary palliative care education (e.g. specialty palliative fellowships, primary palliative skill development for front-line clinicians).

Difficult times invite opportunity. The U.S. healthcare system prides itself on unparalleled leadership in clinical care and research. Now is the time to double down on that commitment and ensure that those facing serious and complex illness–eventually, all of us–receive the excellent care they (and we) deserve, regardless of their state of residence, tax status of their healthcare organization, or other demographics.

## Contributors

Conceptualization—WER; Writing—original draft: WER, DEM, RMA; Writing—review and editing: All authors.

## Declaration of interests

AHK is President, American Academy of Hospice and Palliative Medicine. RMA is Section Editor for Palliative Care, Up-to-Date, Board Member, VitalTalk, and has received honorarium for Teach Like a Champion. DEM is a contributor to Up-to-Date and a Board Member for Courageous Parent's Network and Project Guardianship. The authors have no other disclosures.

## References

[1] (2017). Assessing State variation in high-need adult populations and their care experiences. https://www.commonwealthfund.org/publications/issue-briefs/2017/aug/assessing-state-variation-high-need-adult-populations-and-their.

[2] Kuo D.Z., Houtrow A.J., COUNCIL ON CHILDREN WITH DISABILITIES (2016). Recognition and management of medical complexity. Pediatrics.

[3] Knaul F.M., Farmer P.E., Krakauer E.L. (2018). Alleviating the access abyss in palliative care and pain relief-an imperative of universal health coverage: the Lancet Commission report. Lancet.

[4] Harding R., Hammerich A., Peeler A. (2024). Palliative care: how can we respond to 10 years of limited progress?. Qatar Foundation, World Innovation Summit for Health, World Health Organization. https://wish.org.qa/research-report/palliative-care/.

[5] Connor S.R. (2020). Global Atlas of palliative care, 2nd Ed 2020. http://www.thewhpca.org.

[6] Tripodoro V.A., Fidalgo J.F.L., Pons J.J. (2025). First-ever global ranking of palliative care: 2025 world map under the new WHO framework. J Pain Symptom Manag.

[7] Committee on Approaching Death: Addressing Key End of Life Issues (2015). Institute of medicine. Dying in America: improving quality and honoring individual preferences near the end of life. Washington (DC): National Academies Press (US). http://www.ncbi.nlm.nih.gov/books/NBK285681/.

[8] Snijders R.A.H., Brom L., Theunissen M. (2023). Beuken-van Everdingen MHJ van den. Update on prevalence of pain in patients with cancer 2022: a systematic literature review and meta-analysis. Cancers (Basel).

[9] The Commonwealth Fund, The New York Times, Harvard T.H. Chan School of Public Health (2018). Being seriously ill in America today. https://hsph.harvard.edu/wp-content/uploads/2024/10/CMWF-NYT-HSPH-Seriously-Ill-Poll-Report.pdf.

[10] Pinto S., Lopes S., Sousa A.B., Delalibera M., Gomes B. (2024). Patient and family preferences about place of end-of-life care and death: an umbrella review. J Pain Symptom Manag.

[11] CDC (2019). Trends in inpatient hospital deaths: national hospital discharge survey, 2000–2010. https://www.cdc.gov/nchs/products/databriefs/db118.htm.

[12] Institute of Medicine (2003). When children die: improving palliative and end-of-life care for children and their families. Washington, D.C.: National Academies Press. http://www.nap.edu/catalog/10390.

[13] Yaffe K., Fox P., Newcomer R. (2002). Patient and caregiver characteristics and nursing home placement in patients with dementia. JAMA.

[14] Schulz R., Beach S.R. (1999). Caregiving as a risk factor for mortality: the caregiver health effects study. JAMA.

[15] Sanders J.J., Temin S., Ghoshal A. (2024). Palliative care for patients with cancer: ASCO guideline update. J Clin Oncol.

[16] Kaasa S., Loge J.H., Aapro M. (2018). Integration of oncology and palliative care: a lancet oncology commission. Lancet Oncol.

[17] Iyer A., Akgün K., Bowman B. (2026). A “PalliPulm” framework to improve palliative care education and practice in pulmonary-critical care medicine: an official American thoracic society workshop report. Ann Am Thorac Soc.

[18] Center to Advance Palliative Care and National Kidney Foundation (2025). The case for palliative care in kidney disease. https://www.capc.org/documents/download/1239/.

[19] Chuzi S., Saylor M.A., Allen L.A. (2025). Integration of palliative care into heart failure care: consensus-based recommendations from the heart failure society of America. J Card Fail.

[20] Mafi V.I.P., Soldera J. (2024). Palliative care for end-stage liver disease and acute on chronic liver failure: a systematic review. World J Methodol.

[21] Kluger B.M., Hudson P., Hanson L.C. (2023). Palliative care to support the needs of adults with neurological disease. Lancet Neurol.

[22] Cenzer I., Covinsky K.E., Cross S.H. (2025). Wealth disparities in end-of-life symptom burden among older adults. JAMA Netw Open.

[23] Burgio K.L., Williams B.R., Dionne-Odom J.N. (2016). Racial differences in processes of care at end of life in VA medical centers: planned secondary analysis of data from the BEACON trial. J Palliat Med.

[24] Gerlach L.B., Kales H.C., Kim H.M. (2021). Prevalence of psychotropic and opioid prescribing among hospice beneficiaries in the United States, 2014-2016. J Am Geriatr Soc.

[25] Cea M.E., Reid M.C., Inturrisi C., Witkin L.R., Prigerson H.G., Bao Y. (2016). Pain assessment, management, and control among patients 65 years or older receiving hospice care in the U.S. J Pain Symptom Manage.

[26] Weaver M.S., Shostrom V.K., Kaye E.C., Keegan A., Lindley L.C. (2022). Palliative care programs in children's hospitals. Pediatrics.

[27] Fischer C., Saly E., Berger M., Masel E.K., Simon J. (2026). Economic evaluations in the palliative and end-of-life care settings: a systematic review of existing evidence, methods and quality. Palliat Med.

[28] National Consensus Project for Quality Palliative Care (2018). Clinical practice guidelines for quality and palliative care 4th edition. https://www.nationalcoalitionhpc.org/ncp.

[29] Center to Advance Palliative Care Palliative care facts and stats. https://www.capc.org/documents/download/665/#:%7E:text=billion%20per%20year.-,•,a%20palliative%20care%20team%20today.

[30] Center to Advance Palliative Care (2024). Telehealth to Extend Palliative Care Access to Rural Care Settings. https://media.capc.org/innovation-project-files/Telepalliative_Care_to_Rural_Care_Settings_FINAL.pdf.

[31] Center to Advance Palliative Care (2024). America's readiness to meet the needs of people with serious illness: 2024 serious illness scorecard. CAPC Serious Illness Scorecard. https://scorecard.capc.org/.

[32] ATI Advisory Nonmedical supplemental benefits in medicare advantage in 2024. https://atiadvisory.com/resources/wp-content/uploads/2023/10/ATI-Advisory-PY2024-Nonmedical-Supplemental-Benefits-Chartbook.pdf.

[33] Advanced care planning tool - renal physicians association. https://www.renalmd.org/page/AdvancedCare.

[34] Oncology Nursing Society (2014). Palliative care for people with cancer | Oncology Nursing Society. https://www.ons.org/network-advocacy/position-statements/palliative-care-people-cancer.

[35] Damani T. (2018). Caring for the Hospitalized Child.

[36] Braun L.T., Grady K.L., Kutner J.S., Adler E. (2016).

[37] Renal Physicians Association (2017). Advanced care planning tool. https://www.renalmd.org/page/AdvancedCare.

[38] Buehler N.J., Frydman J.L., Morrison R.S., Gelfman L.P. (2023). An update: national institutes of health research funding for palliative medicine 2016-2020. J Palliat Med.

[39] Advancing the science of palliative care across the lifespan (ASCENT) consortium. NIH Research Grant 1U54 AG093230-01. https://ascentpalliativecare.org/.

[40] Kwon Y., Hu X., Shi K.S. (2025). Contemporary patterns of end-of-life care among medicare beneficiaries with advanced cancer. JAMA Health Forum.

[41] National Hospice and Palliative Care Organization and National Alliance for Care at Home (2024). NHPCO facts and figures 2024 edition. https://allianceforcareathome.org/wp-content/uploads/2024/09/Facts-Figures-2024_FINAL.pdf.

[42] King A., Ortiz C., Goswami R. (2025). Time and location of specialty palliative care for women dying with metastatic breast cancer. Breast Cancer Res Treat.

[43] Kang H.A., Wang B., Barner J.C. (2024). Opioid prescribing and outcomes in patients with sickle cell disease Post–2016 CDC guideline. JAMA Intern Med.

[44] Dowell D. (2022). CDC clinical practice guideline for prescribing opioids for pain — united States, 2022. MMWR Recomm Rep (Morb Mortal Wkly Rep).

[45] Sullender R.T., Selenich S.A. (2016). Financial considerations of hospital-based palliative care. Research Triangle Park (NC): RTI Press. http://www.ncbi.nlm.nih.gov/books/NBK532652/.

[46] Welch WP, Xu L, Lew ND, Sommers BD. Ownership of Hospitals: An Analysis of Newly-Released Federal Data & A Method for Assessing Common Owners. 40700551

[47] Stevenson D., Peterson H., Skinner R. (2023).

[48] Kannan S., Bruch J.D., Song Z. (2023). Changes in hospital adverse events and patient outcomes associated with private equity acquisition. JAMA.

[49] Soltoff A.E., Unruh M.A., Stevenson D.G., Kavalieratos D., Braun R.T. (2024). Caregiver-reported quality in hospices owned by private equity firms and publicly traded companies. JAMA.

[50] Preying on the Dying (2023). Private equity gets rich in hospice care. CEPR. https://cepr.net/publications/preying-on-the-dying-private-equity-gets-rich-in-hospice-care/.

[51] Morrison R., Penrod J., Cassel J. (2008). Cost savings associated with US hospital palliative care consultation programs. Arch Intern Med.

[52] Temel J.S., Greer J.A., Muzikansky A. (2010). Early palliative care for patients with metastatic Non–small-cell lung cancer. N Engl J Med.

[53] Cuevas -Karina (2025). Millions of seniors lose access to telehealth services in wake of shutdown. PBS News. https://www.pbs.org/newshour/show/millions-of-seniors-lose-access-to-telehealth-services-in-wake-of-shutdown.

[54] kffcarenec (2026). What to know about medicare coverage of telehealth. KFF. https://www.kff.org/medicare/what-to-know-about-medicare-coverage-of-telehealth/.

[55] Greer J.A., Temel J.S., El-Jawahri A. (2024). Telehealth vs In-Person early palliative care for patients with advanced lung cancer: a multisite randomized clinical trial. JAMA.

[56] Kamal A.H., Wolf S.P., Troy J. (2019). Policy changes key to promoting sustainability and growth of the specialty palliative care workforce. Health Aff.

[57] (2023). Center to advance palliative care and the palliative care quality collaborative. Spotlight on Home-Based Palliative Care. https://www.capc.org/documents/download/1100/.

[58] Aldridge M.D., Kelley A.S. (2015). The myth regarding the high cost of end-of-life care. Am J Public Health.

[59] (2024). Mirror, Mirror 2024: A portrait of the failing U.S. health system. https://www.commonwealthfund.org/publications/fund-reports/2024/sep/mirror-mirror-2024.

[60] Gunja M., Gumas E., Williams R. (2023).

[61] Hollenbach F., Szakonyi D. (2025).

[62] Center for Health Care Strategies The better care playbook is now the evidence-to-action hub!. https://www.chcs.org/the-better-care-playbook-is-now-the-evidence-to-action-hub/.

[63] Kathleen K. (2018). Palliative care in California: narrowing the gap. https://www.chcf.org/wp-content/uploads/2018/05/NarrowingGap.pdf.

